# Mobile Breast Cancer e-Support Program for Chinese Women With Breast Cancer Undergoing Chemotherapy (Part 1): Qualitative Study of Women’s Perceptions

**DOI:** 10.2196/mhealth.9311

**Published:** 2018-04-11

**Authors:** Jiemin Zhu, Lyn Ebert, Dongmei Guo, Sumei Yang, Qiuying Han, Sally Wai-Chi Chan

**Affiliations:** ^1^ Nursing Department Medical School Xiamen University Xiamen China; ^2^ School of Nursing and Midwifery Faculty of Health and Medcine University of Newcastle Newcastle Australia; ^3^ Zhongshan Hospital Xiamen University Xiamen China

**Keywords:** mobile app, breast cancer, chemotherapy

## Abstract

**Background:**

Women with breast cancer undergoing chemotherapy experience difficulty in accessing adequate cancer care in China. Mobile apps have the potential to provide easily accessible support for these women. However, there remains a paucity of randomized controlled trials to evaluate the effectiveness of app-based programs targeting specifically women with breast cancer undergoing chemotherapy. Moreover, women’s perceptions and experiences related to using and interacting within the app-based program have rarely been reported. Therefore, an app-based Breast Cancer e-Support program was developed and evaluated using a randomized controlled trial. Based on the incorporation of Bandura’s self-efficacy and social exchange theory, Breast Cancer e-Support program lasted for 12 weeks covering 4 cycles of chemotherapy and had 4 components: (1) a Learning forum, (2) a Discussion forum, (3) an Ask-the-Expert forum, and (4) a Personal Stories forum.

**Objective:**

As a part of the randomized controlled trial, the aim of this study was to explore the participants’ perception of Breast Cancer e-Support program, its strengths and weaknesses, and suggestions to improve the program.

**Methods:**

A descriptive qualitative study was employed. Thirteen women with breast cancer from 2 university-affiliated hospitals in China, who were randomly allocated to the Breast Cancer e-Support program in the randomized controlled trial, were interviewed from November 2016 to February 2017. Purposive sampling was used based on women’s scores of self-efficacy after the completion of the intervention. Inductive content analysis was used to analyze the transcripts, allowing the categories and subcategories to flow from the data.

**Results:**

The qualitative interviews revealed that participants perceived the Breast Cancer e-Support program to be helpful in enhancing knowledge, improving confidence level, and promoting emotional well-being. Women also identified access to tailored advice from experts and convenience as the benefits of this program. Physical or psychological health status, stigma related with breast cancer, and app instability were mentioned as the challenges to engagement. Suggestions for improvement included adding message reminders to prompt instant communication and search engine to locate information quickly, supplementing more interesting and practical knowledge, updating the information more often, and quickening the responses to women’s questions. The participants recommended the Breast Cancer e-Support program to be incorporated as routine care to support women during chemotherapy.

**Conclusions:**

This study demonstrates the potential of the Breast Cancer e-Support program to support women during chemotherapy. Future app-based programs should apply a family-centered approach and provide more support on stigma associated with the disease to encourage engagement with the app. Suggestions of improvement regarding the design, content, and operation of the app-based intervention should be addressed in future studies. It is promising to incorporate the Breast Cancer e-Support program into routine care to generalize the benefits.

**Trial Registration:**

Australian New Zealand Clinical Trials Registry ACTRN12616000639426; http://www.ANZCTR.org.au/ ACTRN12616000639426.aspx (Archived by WebCite at http://www.webcitation.org/6v1n9hGZq)

## Introduction

Breast cancer is the most commonly diagnosed cancer for Chinese women [1], and chemotherapy is widely used to treat breast cancer [2]. Women with breast cancer receiving chemotherapy experience difficulty accessing adequate cancer care in China because of the shortage of oncology-trained health care professionals (HCPs) [3,4], and the increasing incidence of breast cancer [5]. In recent years, China’s Ministry of Health has developed a national plan aiming to provide easily accessible and affordable health service to prevent and control cancer [6]. Mobile apps offer one means in this endeavor to support patients [7].

It is widely recognized that the use of chemotherapy causes a variety of side effects such as nausea, insomnia, and pain, which adversely affect women’s psychological well-being and quality of life (QoL) [8,9]. Effective symptom management is thus crucial, and a sense of self-efficacy and social support are needed for women to initiate and maintain appropriate symptom management strategies [10]. Furthermore, the majority of women receiving chemotherapy for breast cancer are treated in the out-patient setting and have to manage most symptoms at home without direct support from HCPs [11]. Apps could provide an innovative platform to overcome the accessibility barrier, where women can acquire knowledge and communicate with peers and HCPs when and where needed [7].

In 2017, 89% of the Chinese population owned a mobile phone [12], and approximately 653 million Chinese people accessed the internet via their mobile phone [13]. Taking advantage of their easily accessibility, apps have the potential to demonstrate their value on health promotion with a robust program. However, our recent integrative review found that there remains a paucity of randomized controlled trials (RCTs) to evaluate the app effectiveness specifically targeting at women with breast cancer undergoing chemotherapy [14]. Furthermore, women’s perceptions and experiences related to using and interacting within the app-based programs during chemotherapy have rarely been reported [14].

Therefore, an app-based Breast Cancer e-Support program was developed [15] and evaluated using a single-blinded, multicentered RCT (ACTRN: ACTRN12616000639426) [16]. The components of this program were guided by four factors (direct mastery experiences, vicarious experiences, verbal persuasion, and arousal state) from Bandura’s self-efficacy theory [17] and structural support and functional support from the social exchange theory [18]. This program supported women for 12 weeks covering four cycles of chemotherapy and had been shown to significantly improve the self-efficacy, QoL, and symptom interference on daily life for women at 3 months of follow-up, as reported in Part 2 of this study (forthcoming) [19].

Process evaluation is an important part of an RCT to understand the program effectiveness [20]. Identifying how women appraise different components, especially for a multicomponent app-based program, can give insights as to why and how the program achieves or fails in the desired outcomes, further improving the design of a future trial [21]. Thus, this qualitative process evaluation aimed to explore the participants’ perception of Breast Cancer e-Support program, the strengths and weaknesses of this program, as well as their improvement suggestions.

## Methods

### Study Design and Participants

A descriptive qualitative study was employed. The first author recruited women from the intervention group in an RCT examining the effectiveness of the Breast Cancer e-Support program. Women were eligible for the main study if they had commenced chemotherapy at the study sites after diagnosis of breast cancer, were able to access the internet via a mobile phone, and were able to read and write Mandarin. The study was approved by the institutional review board of Xiamen University affiliated Zhong Shan Hospital (zhu20151023), Central South University affiliated Hunan Cancer Hospital (zhu20151026) in China, and the University of Newcastle in Australia (H-2015-0448). Written consent forms were obtained from all participants.

### Breast Cancer e-Support Program

**Figure 1:**
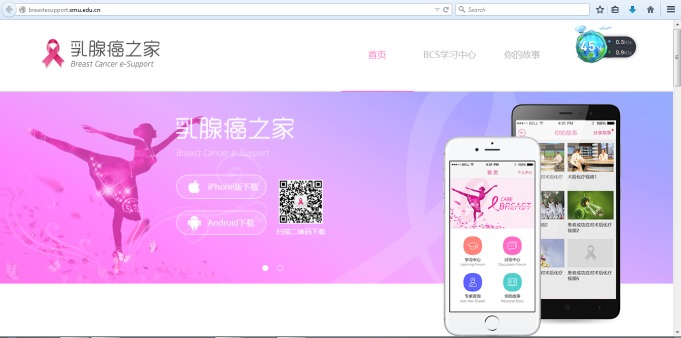
Screenshot of the Breast Cancer e-Support program Website.

A website was developed, with the introduction of the Breast Cancer e-Support program and the two-dimensional code for women to scan (Figure 1) [22]. Once women downloaded the app, the home page displayed a button for the user to submit an application that the first author (JZ) either approved or declined through the app background thread to ensure only the intervention group had access to the Breast Cancer e-Support program with a unique username and self-set password. This application process prevented contamination between the two groups and protected the privacy of participants in the Breast Cancer e-Support program.

The development process of the Breast Cancer e-Support program applied user-centric design and assessed the perceived ease of using the app [15]. This program supported women for 12 weeks covering four cycles of chemotherapy (1st-4th cycle of chemotherapy), with each cycle lasting 3 weeks. The Breast Cancer e-Support program had four components: Learning forum, Discussion forum, Ask-the-Expert forum, and Personal Story Forum [16]. The Learning forum provided knowledge and symptom management strategies related to breast cancer and chemotherapy. The Discussion forum offered an online platform for women to chat with one another. The Ask-the-Expert forum served as a communication channel for women to ask questions and receive advice directly from experts within 24 hours. The Personal Story forum presented five encouraging stories of other women who had successfully overcome obstacles during chemotherapy. On the basis of women’s topics and questions, the information in the learning forum was updated every 2 weeks to address women’s concerns. The Discussion forum and Ask-the Expert forum were moderated by the first author (JZ) who is an HCP. The moderator facilitated the online discussions and sent a reminder message to the doctors with incoming questions. Eight doctors from the study sites joined this program and responded to the questions in the Ask-the Expert forum. Technical assistance was available for the workday. The details of the Breast Cancer e-Support program can be found in the study protocol published in *BMC Cancer* [16].

### Recruitment

For the process evaluation, we applied purposive sampling based on scores in the primary outcome of self-efficacy at 3 months. The Stanford Inventory of Cancer Patient Adjustment was used to assess women’s self-efficacy to manage cancer-related problems [23]. The Stanford Inventory of Cancer Patient Adjustment contained 38 items rated from 0 (not at all confident) to 10 (absolutely confident). Higher total scores represent higher self-efficacy. We selectively approached women with higher and lower (ratio 1:1) scores than the mean score of self-efficacy. We achieved data saturation at the 10th participant. No additional data were yielded with three additional interviews, giving a total sample of 13 women [24]. Of the 13 interviewed women, 7 participants were higher and 6 participants lower than the mean score of self-efficacy.

Interviews were conducted from November 2016 to February 2017. As these two participating hospitals are in two different provinces in China, we undertook face-to-face interviews for women in Zhong Shan Hospital and telephone interviews for women in Hunan Cancer Hospital to achieve a sufficient sample size. The first author (JZ), who was a female PhD student in nursing and who had established a good relationship with women during moderating the online discussion, invited women by telephone to participate, giving a clear explanation of the aims of the interview. Women were informed that the interview would take 10 to 20 min and that the interview would be audiorecorded. A total of 18 women were approached. Five women declined to be audiorecorded and thus excluded. A total of 5 women from Zhong Shan Hospital were interviewed one-to-one in a meeting room in the hospital. Field notes were taken during the interviews. A total of 8 women from Hunan Cancer Hospital were interviewed by telephone. The duration of the interviews ranged from 7 to 27 min.

A semistructured interview guide (Table 1) with five questions was prepared for the interview. Two academic experts in psychoeducation and cancer care provided comments on clarity and content of the interview guide.

**Table 1:** Semistructured interview guideline for process evaluation.

No.	Probing questions
1	What was your experience of using this program?
	Probe: How did you feel participating in this program?
2	What were the main strengths or benefits of this program for you?
	Probe: Which aspect of this program was most beneficial for you? Why?
3	What were the main weakness of this program for you?
	Probe: Which aspect of the program was least beneficial for you? Why?
4	What suggestion will you give to improve this program?
	Probe: How can this program be improved further?
5	Do you think this program should be continued?

### Analysis

Inductive content analysis was used to analyze the transcripts, allowing the categories to flow from the data [25]. Qualitative content analysis was conducted in the original language, following Graneheim and Lundman’s [26] steps for analysis procedures to achieve trustworthiness. Audiotapes were transcribed verbatim into written Chinese by the first author immediately after the interview. The first (JZ) and third authors (DG) performed the initial coding independently. Transcripts and field notes were repeatedly read through to obtain a broad picture of the whole. Transcribed data were coded and analyzed. The various codes were compared regarding the differences and similarities, and interpretations of the codes were sorted into subcategories. Finally the underlying meanings of the subcategories were grouped into categories. The research team had regular meetings to examine and revise the tentative subcategories and categories. Any coding discrepancies were discussed to ensure consensus. Two experts in qualitative research reviewed the process of data collection and analysis. Participant’s quotes were translated into English. English translation of the results and direct quotes were checked by the last author who is bilingual (English and Chinese).

## Results

### Sample Characteristics

For all 114 participants in the RCT, the mean of self-efficacy score at 3 months was 212.1 (SD 58.1; range 74-368). The mean age was 47.2 years. The majority of women were married (96.5%, 110/114), had received elementary or middle school (57%, 65/114) education, and were currently unemployed (74.6%, 85/114). Only 14 women (12.3%, 14/114) lived in a household with above average level of monthly family income (≥US $739) [27]. The majority of women were diagnosed with breast cancer stage II (43.0%, 49/114), followed by stage III (36.7%, 42/114). A total of 97 women (85.1%, 97/114) had undergone a mastectomy, and 5 (4.4%, 5/114) had chosen breast conserving surgery. The majority of women received eight cycles (51.8%, 59/114) and six cycles (21.1%, 24/114) of chemotherapy.

In total, 13 women in the intervention group participated in the interview, with mean score of self-efficacy at 3 months as 218.58 (SD 68.1; range 108-349). These 13 women had comparable demographic or clinical characteristics as all participants in the RCT, except for education level (χ^2^_0.05,4_=10.8, *P*=.01). The interviewed women had significantly higher education level than all participants in the RCT. The demographic or clinical variables of the 13 participants are summarized in Table 2.

### Overview

From the content analysis, four categories emerged: (1) benefits of participation, (2) challenges to engagement, (3) suggested improvement, and (4) future direction. All the categories and subcategories are summarized in Textbox 1.

#### Category 1: Benefits of Breast Cancer e-Support Program

A total of 9 women found that the Learning forum was most beneficial, and 3 believed that the Discussion forum was most beneficial. A total of 5 women found the Personal Story forum as being least beneficial. Six women did not identify any forum to be least beneficial. Participants reported many benefits from the different forums, including enhanced knowledge, improved confidence levels, promoted emotional well-being, access to tailored advice from experts, as well as being easy to use, convenient, and easily accessible.

##### Subcategory 1: Enhancing Knowledge on Symptom Management, Breast Cancer, and Chemotherapy

Most of the women mentioned that they gained a better understanding of breast cancer, chemotherapy, and how to manage a variety of symptoms after participating in the Breast Cancer e-Support program:

I was very interested in reading the content in the Learning forum. In the past, I never thought about breast cancer. Before the diagnosis, I had no knowledge on anything about breast cancer...But now it happened to me. I read the knowledge in the Learning forum and I really have learnt a lot. I felt good when reading the content.

Informant 7

I like to read information on symptom self-management. In the past, I searched the web for it, but was not sure whether the information was useful. I trust the information on the app to be accurate...You know, when I was having chemotherapy after surgery, I was a bit scared. I did not know what symptoms I would experience, how to self-manage symptoms, how to choose food, and how to prevent oedema. The Learning forum has everything...the related information. I learnt a lot. I obtained knowledge which I did not know before.

Informant 5

##### Subcategory 2: Improved Confidence Level

Two women said they felt more confident as they watched the encouraging videos in the “Personal Story forum,” and 3 women mentioned that their confidence levels were increased when reading other participant’s experiences shared in the “Discussion forum.” Others’ stories served as the role models to build confidence for these women. When women saw someone succeed in a similar situation, they felt empowered and more positive:

My favourite is “Personal Story forum”. They are real stories. I like to watch others’ stories. For example, Miss XX, who is 60 years old, wear a wig, put on make-up, and travel a lot after chemotherapy. I think attitude is very important. Miss XX is older than me and had more advanced breast cancer. When I saw she could survive the chemotherapy and had a good life, I felt more confident that I can do it.

Informant 3

**Table 2:** Demographic or clinical data of participants in the process evaluation.

Items		Value
Age in years, mean (SD; range)		49.5 (9.5; 30-65)
**Married status, n (%)**		
	Married	13 (100)
**Education level, n (%)**		
	University level or above	4 (31)
	High school	6 (46)
	Middle school	2 (15)
	Primary school	1 (8)
**Current employment, n (%)**		
	Employed	3 (23)
	Unemployed	10 (77)
**Monthly family income (USD), n (%)**		
	≥$739^a^	3 (23)
	$443-$738	5 (39)
	$149-$442	3 (23)
	≤$148	2 (15)
Body mass index, mean (SD; range)		23.3 (2.3; 19.5-27)
**Cancer stage, n (%)**		
	Stage III	6 (46)
	Stage II	4 (30)
	Stage I	3 (23)
**Surgery, n (%)**		
	Mastectomy	12 (92)
	Breast conserving surgery	1 (8)
**Cycles of chemotherapy, n (%)**		
	Eight cycles	6 (46)
	Six cycles	4 (31)
	Four cycles	3 (23)

^a^≥US $739 above average level of monthly family income in China [27].

**Categories and subcategories from the process evaluation.**

Benefits of Breast Cancer e-Support program

- Enhanced knowledge
- Improved confidence level
- Improved emotional well-being
- Received advices from experts
- Easy to use, easily accessible, and convenient

Challenges to engagement of Breast Cancer e-Support program

- Physical or psychological health status
- Stigma with breast cancer
- Instability of the app

Suggested improvement

- Design improvement
- Interesting, plain, and practical content
- The information being updated more often
- Quicker responses to women’s questions

Future direction

- Breast Cancer e-Support program as routine care
- Breast Cancer e-Support program open to caregivers
- Breast Cancer e-Support program applied to other cancer patients

##### Subcategory 3: Improved Emotional Well-Being

Most women mentioned that they felt much better when they read the information in the Learning forum and interacted with peers and HCPs through the program. They were less distressed as their perception about breast cancer altered and their queries answered. They felt reassured knowing that they were not alone in the struggle with breast cancer, and they had peer and professional support available. This is revealed from the following statements by 2 women:

I feel better to talk to someone who are in similar situation. Cancer is not a good thing. If I always think about breast cancer alone at home, it is so easy for me to feel bad. I didn’t feel alone when I talked with peers through your program. They might have worse or better conditions than me, but they understand what I meant (Laugh...). This may be the source of comfort and help.

Informant 13

Before I joined your program, I searched online and I was overwhelmed by the horrible information on breast cancer. I got more and more anxious during surfing the internet. But your program was different. There was credible information and expert advice. The more I used the app, the better I felt.

Informant 2

##### Subcategory 4: Received Advice From Experts

Some women appreciated the Ask-the-Expert forum as the advice was tailored to their own needs, which was “direct and targeted help.” They felt supported as they had experts to answer their queries and received timely responses. The advice from an expert acknowledged their symptoms, validated their concerns, interpreted the lab results, and gave some useful suggestions. One woman stated:

When I faced with something I didn’t know, I was so anxious. But my doctor was very busy and he had no time to communicate with me. After I jointed your program, I could ask questions through the app regarding my medical condition. I could upload the lab results through your program. Then I received corresponding advice from experts. I felt followed up. When I knew more about my medical condition, I felt more likely to gain control of my life.

Informant 4

##### Subcategory 5: Easy to Use, Easily Accessible, and Convenient

Ten women expressed that one of the biggest benefits of the Breast Cancer e-Support program was that it was easy to use and convenient. Tapping into the app enabled them access to the knowledge and easy communication with peers and HCPs. This was especially helpful for women who lived long distances from the hospital. One woman stated:

I have no difficulty in using the app. I live far away from the hospital and I have no doctor close to me. When I had questions about my medical condition, I could not find the answer in the internet. Then I asked questions through the app. Aha, the professor or expert responded. Sometimes they gave me quick feedback. Sometimes, they answered my questions the next day. Yes, we can also make our judgement, but we are not sure at that time. The response from the expert provided me the direction. I believe this is the strength of the app.

Informant 8

#### Category 2: Challenges to Engagement of Breast Cancer e-Support Program

Participating women highlighted several challenges to engagement in the Breast Cancer e-Support program. Challenges were associated with physical or psychological health status, stigma with breast cancer, and instability of the app.

##### Subcategory 1: Physical or Psychological Health Status

Some women mentioned that the severe physical symptoms such as nausea and fatigue hindered their engagement of the program. Psychosocial symptoms related to fear of recurrence and death added to their distress, further hampering women’s engagement of Breast Cancer e-Support program. This is echoed by the following statements:

During the three days hospitalization for chemotherapy, I felt like dying and I couldn’t even think about opening the app. When I came back home and I recovered a little bit, still my health was quite fragile. I couldn’t spend long time reading the app or have enough energy to read in depth.

Informant 1

During chemotherapy, I guess, all people felt very down. We had lots of negative thoughts (Pause for a while). For me, I would reduce the usage of the app. When I recovered a little bit, I might resume using the program.

Informant 2

##### Subcategory 2: Stigma With Breast Cancer

Some women experienced stigma with breast cancer. They perceived themselves as having a “disability” and felt that they would be rejected by family and friends if they made their condition known. Women felt ashamed to have breast cancer, and using this program reminded them of their illness. Women who felt this way reduced the usage of the Breast Cancer e-Support program because they did not want to be reminded that they had breast cancer or they were different:

If I told my friends that I had breast cancer, they would reject me. I had such experience...They perceived me as a different person. How can I have the courage to tell people about my disease? I do not want to touch the topic of “breast cancer”. I’ve tried to put it behind me...Using this program, reading and chatting, it constantly reminds me of my illness. I need to be done with it.

Informant 7

##### Subcategory 3: Instability of the App

One woman complained that the app was not stable. She gave up using the program when experiencing difficulties in logging in. She stated:

The app sometimes was unstable. It didn’t work when I tried to open it. I contacted with someone in the hospital and reinstalled the app. Then I could log in. However, after a periods of time, I couldn’t open the app again. Finally I gave up using your program. I haven’t log in for the recent month.

Informant 5

#### Category 3: Suggested Improvement

Many women gave suggestions on how to improve this program, including design improvement; interesting, plain, and practical content; updating the information more often; and quickening response to women’s questions.

##### Subcategory 1: Design Improvement

Some women suggested a design or technical improvement such as adding the message reminder and search engine to facilitate instant communication in the Discussion forum and help women to locate the information they needed in the Learning forum. Two women were of the following opinion:

The app may be improved by adding the message reminder, like that in chat tools such as QQ or Webchat. Once someone writes a message, the app will have message reminder to notify others. Then others will read and join the discussion..., like conversation with each other. If you write a message and nobody responds, then you lose the interest to continue writing. Sometimes we just need to talk to others. But if we always need wait for a while, we may lost interest.

Informant 2

There are too many content in the Learning forum. I was overwhelmed by the information each time I opened it. I do not have patience to read all of them...But the screen of the mobile phone is so small and it takes long time to find the knowledge you want. The program can be improved by adding search engine in the Learning forum. If I search for “nausea”, then all the knowledge related to nausea will come out. Search engine will help save my time.

Informant 3

##### Subcategory 2: Short, Interesting, Plain, and Practical Content

Some women mentioned that the information in the Leaning forum should be short, interesting, and practical. Also plain language should be used to convey the knowledge. Adding more content on food choice was requested by 6 women. Five women said they didn’t tap into the “Personal Story forum” very often and hadn’t benefitted from the video much. Videos being too long was identified as the main reason, and the majority of women suggested that the video should be short and focused:

Please add more information on the food choice. We need to eat every day, however, there is conflicting advice on food choices on the internet, such as whether we should eat honey, chicken, leek, etc. We are in a dilemmaon what we should eat. The apps can provide detailed information on food choice, the time of food intake, the cooking methods, etc...Such practical information would be very helpful.

Informant 11

The videos are always long. I opened the first video and it showed that the video would took 25 minutes. I thought I would watch it when I had enough time. Then I never found time to open it again. I forgot it (Laugh...). Actually, when I was quite physically fragile, I prefer to short and fast information...sometimes, fragmental information. I like to use the Ask-the-Expert forum and Learning forum to search for answers to my own questions. It was quick. I couldn’t settle myself down to watch the whole video. The video should be short and focused. Three to five minutes would be enough for one story.

##### Subcategory 3: Update the Information More Often

Some women mentioned that the information should be updated more often because they required different information and faced new problems as their chemotherapy progressed. One woman stated:

We have so many people in the discussion forum. En...During chemotherapy, always there are new problems occurred for different people. Reading the same materials seems inadequate. We hope there are more updated information. For me, I have no illness experience before and I have no medical knowledge. Every day I like to open the Learning forum for reading. It will be great if you could update more often your information such as food choice, or medical follow-up.

Informant 7

##### Subcategory 4: Quicken the Responses to Women’s Questions

Some women said they preferred receiving an answer immediately from the HCPs, but there were often delays in responses to their questions:

I like the Ask-the-Expert forum. But, if we have some questions in my mind, we are quite anxious. I want a response right away. But, it seems...maybe the doctor were at rest, or at work. It always took some time for the doctors to answer our questions.

Informant 11

#### Category 4: Future Direction

Most women were satisfied with this program and advocated it as routine care. Some women also suggested opening this program to caregivers. One woman recommended applying this program to other cancer groups.

##### Subcategory 1: Breast Cancer e-Support as Routine Care

Most of the women said that as this program was very beneficial for them, this program should be available to more women with breast cancer as routine care:

It is great if all women with breast cancer could receive this program, which might help them go through the chemotherapy. Thank you very much...From my own experience in this program, I knew more about breast cancer and chemotherapy, and I felt more confident to copy with the disease. The programs must be helpful for them you know.

Informant 1

##### Subcategory 2: Breast Cancer e-Support Open to Other Family Members

Some women suggested that the Breast Cancer e-Support program should be open to other family members, especially for women who are illiterate and who are very old:

Many women with breast cancer come from the countryside. They are illiterate, or they cannot read and speak Mandarin. However, they also suffered a lot from the chemotherapy. If you can open the program to other family members who can read and convey the knowledge to the women, they would also benefit from your program.

Informant 2

Some people, like me, 40 or 50 years old. Well, this group believe the apps is a little bit troublesome. They feel challenged to use the new technology. This is a problem. Although they are not willing to participate, they often consulted me on some questions and they were quite interested in the knowledge. If this program can be available for their family members, such as their son or daughter, it would be helpful.

Informant 11

##### Subcategory 3: Breast Cancer e-Support Applied to Other Cancer Patients

One woman, who actively participated in the Discussion forum and Ask-the Expert forum, mentioned that this program should be applied to all cancer patients because of common issues such as health, food choice, and exercise. She stated:

If the app can be used by all types of cancer patients, not only breast cancer, which will be good. We have lots of common topics to discuss, such as maintaining health, choosing appropriate food, exercising on a regular basis. That will be a great platform to promote health for cancer patients. From my point of view, it is quite helpful to expand the scope of the application. Haha...

Informant 6

## Discussion

### Principal Findings

In this study, we evaluated participants’ perception of an app-based program that they had access to during four cycles of chemotherapy. This process evaluation supported the quantitative results of the RCT that the Breast Cancer e-Support program was useful and feasible [19]. This study helped in understanding what challenges exist with the engagement of the Breast Cancer e-Support program. Despite the challenges, the participants provided constructive suggestions for improvement related to the design, content, and operation of this program. The participants recommended the Breast Cancer e-Support program be incorporated as routine care to support women during chemotherapy.

Women in this study reported that the Breast Cancer e-Support program was helpful in enhancing knowledge, improving confidence level, and promoting emotional well-being. They valued the tailored advice from experts. These findings could be explained by the theoretical framework of this program: Bandura’s self-efficacy theory [17] and the social exchange theory [18]. Self-efficacy, an individual’s perceived ability to perform a particular task in a given situation, is an important concept that influences women’ ability to manage their disease and chemotherapy [17]. Four factors such as direct mastery experiences, vicarious experiences, verbal persuasion, and perception of their physiological state from Bandura’s self-efficacy theory [17] were addressed in the Breast Cancer e-Support program by offering knowledge and skills, providing hopeful stories, and encouragement from peers and HCPs. In addition, supported by social exchange theory, the Breast Cancer e-Support program provided a channel to connect women with structural support and a variety of functional support, which are identified as the essential components of social support [18].

Many women stated that the Breast Cancer e-Support program was easy to use and convenient. Compared with traditional face-to-face interventions, connecting with the Breast Cancer e-Support program meant that women were able to access information and social support at anytime and anywhere [28]. Taking advantage of the app’s technical capabilities, this program provided women the opportunity to learn tailored information, watch encouraging stories, and chat with peers and HCPs [16]. Even though the majority of women enjoyed the convenience and multifunctional ability of the Breast Cancer e-Support program, one woman identified the app instability as a challenge to the engagement of this program. It is hard to determine whether the app instability was because of the internet connection or the app itself. But still, there is a need to improve the app reliability and to offer sufficient technical support service to participants [29].

This study revealed that some participants felt too sick or in a low mood to use the Breast Cancer e-Support program during chemotherapy. Consistent with our study, the association between poor health status and infrequent computer use was reported [29,30]. Furthermore, Chinese women might fail to disclose their negative emotions when confronting a life-threatening disease [31], which may have prevented participants in this study from sharing their feelings in the Breast Cancer e-Support program. In traditional Chinese culture, family members are obliged to take care of the patients [32]. Family-centered approach should therefore be adopted in the future app-based studies, so that a family member can also use the app in need when women are experiencing severe physical and psychosocial symptoms.

In our study, stigma related to breast cancer was reported to prevent the utilization of this program. Culturally, Chinese women are not willing to talk openly about breast cancer because of the perceived social rejection and low self-esteem associated with being ill and the disfigurement of breast surgery [31]. Women’s concerns on stigma may also hinder their emotional expression and support seeking behaviors in this program, thus reducing their engagement. Culturally sensitive education and peer support were reported to help reduce stigma and increased a sense of belonging for Chinese women with breast cancer [33]. Integration of culturally tailored support regarding stigma into the Breast Cancer e-Support program may alter women’s perception on stigma, thus improving their engagement.

During process evaluation, women also gave constructive suggestions for improvement in design, content, and operation of the Breast Cancer e-Support program. Design improvement involved adding message reminders to prompt instant communication with peers and shorten the response time from the experts, as well as adding a search engine function to help quickly locate information. Content improvement included supplementing more interesting and practical knowledge (eg, more pictures, cartoons, and food choices), plain language used, and short and concise videos to convey information. Our study indicates the demand for health service that optimizes on instant communication and improves efficiency on information delivery [34]. Operational improvement referred to knowledge being updated more often and questions being responded to more quickly. HCPs’ involvement, such as knowledge updating and timely feedback, is crucial to reinforce women’s app engagement [34]. In recent years, user-centered design has gained recognition in app development [35], and user-friendliness has been incorporated in the app assessment [36,37]. Thus, these valuable comments from users’ experiences identified in our study should be addressed in future app designs and operation to encourage app engagement.

In our study, half of the interviewed women emphasized the importance of diet guidelines during chemotherapy. They believed that food choice was very practical, and this program should add more diet details. Diet is imbued with Chinese culture [38], and the need for more specific diet information was considered important. Traditional Chinese medicine is evident regarding diet and food properties among Chinese women with breast cancer undergoing chemotherapy [39]. Specific food recommendations that are culturally sensitive should be addressed for future app-based programs targeting different sociocultural groups.

Many participants recommended the incorporation of this program as routine care during chemotherapy. Some suggested the Breast Cancer e-Support program should be generalized to other family members or other cancer patients. These recommendations indicated that women regarded this program useful and beneficial to other patients. With the increasing growth of mobile phone users [12], it is possible for all patients and their family caregivers to have such an app service available. Moreover, benefiting from the rapid advances in mobile technologies [7], future app-based intervention may convey information via multimedia for illiterate and old patients.

### Strengths and Limitations

This study has limitations. This study was limited to those who were able to access the internet via a mobile phone and who were willing to be interviewed. This inclusion criteria may have narrowed the participant cohort to those who were technologically comfortable with mobile phone use and who had a high level of motivation to share their experiences. Moreover, there might be bias of the findings as the interviewed women were better educated than all participants in the RCT. Our results may not be representative of the views of all women with breast cancer in general. Two women in the intervention group who never accessed the program were not interviewed (one lost contact, one refused). Therefore, we were unable to explore further the reasons for nonuse of the app-based program. The strength of the study is that this process evaluation gathered rich qualitative data of participants’ views on the benefits, challenges, improvement suggestions, and recommendation for an innovative intervention.

### Conclusions

The study provides evidence on the benefits of the Breast Cancer e-Support program and contributes to an increased understanding of some challenges of why women could not engage in this program as much as expected. This study also discussed some improvement suggestions and future direction of the app-based program from participants’ view. Such knowledge is crucial, and future app-based interventions could address participants’ improvement suggestions on design and operation, apply family-centered approaches, and provide more support on stigma associated with the condition to encourage the app engagement and provide better support.

Our study also has global significance because apps are being increasingly recognized as supplementary interventions when the feasibility of traditional face-to-face interventions are challenged. This app has the potential to be used by Chinese women in Australia and also be translated to other languages to help the culturally and linguistically diverse groups to promote their health outcomes. The knowledge generated from the study can be used to develop guidelines for future health care app development.

## Abbreviations

HCP: health care professional
QoL: quality of life
RCT: randomized controlled trial
